# Emergence and clonal dissemination of KPC-3-producing *Pseudomonas aeruginosa* in China with an IncP-2 megaplasmid

**DOI:** 10.1186/s12941-023-00577-z

**Published:** 2023-04-29

**Authors:** Haoyu Ge, Jie Qiao, Jiahao Zheng, Hao Xu, Ruishan Liu, Junhui Zhao, Ruyan Chen, Chenyu Li, Xiaobing Guo, Beiwen Zheng

**Affiliations:** 1 Collaborative Innovation Center for Diagnosis and Treatment of Infectious Diseases, State Key Laboratory for Diagnosis and Treatment of Infectious Diseases, College of Medicine, the First Affiliated Hospital, Zhejiang University, Hangzhou, China; 2 Department of Laboratory Medicine, The First Affiliated Hospital of Zhengzhou University, Zhengzhou, China; 3 Institute of Animal Quarantine, Chinese Academy of Inspection and Quarantine, Beijing, 100176 China; 4 Department of Structure and Morphology, Jinan Microecological Biomedicine Shandong Laboratory, Jinan, Shandong China; 5 Research Units of Infectious Diseases and Microecology, Chinese Academy of Medical Sciences, Beijing, China; 6 School of Basic Medical Sciences, Zhejiang Chinese Medical University, Hangzhou, China

**Keywords:** *Pseudomonas aeruginosa*, Carbapenemase, *Bla*_KPC-3_, IncP-2 megaplasmid, ST1076, Clonal dissemination

## Abstract

**Background:**

Despite the global prevalence of *Klebsiella pneumoniae* Carbapenemase (KPC)-type class A β-lactamases, occurrences of KPC-3-producing isolates in China remain infrequent. This study aims to explore the emergence, antibiotic resistance profiles, and plasmid characteristics of *bla*_KPC-3_-carrying *Pseudomonas aeruginosa*.

**Methods:**

Species identification was performed by MALDI-TOF-MS, and antimicrobial resistance genes (ARGs) were identified by polymerase chain reaction (PCR). The characteristics of the target strain were detected by whole-genome sequencing (WGS) and antimicrobial susceptibility testing (AST). Plasmids were analyzed by S1-nuclease pulsed-field gel electrophoresis(S1-PFGE), Southern blotting and transconjugation experiment.

**Results:**

Five *P. aeruginosa* strains carrying *bla*_KPC-3_ were isolated from two Chinese patients without a history of travelling to endemic areas. All strains belonged to the novel sequence type ST1076. The *bla*_KPC-3_ was carried on a 395-kb IncP-2 megaplasmid with a conserved structure (IS*6100*-IS*Kpn27*-*bla*_KPC-3_-IS*Kpn6*-*korC*-*klcA*), and this genetic sequence was identical to many plasmid-encoded KPC of *Pseudomonas* species. By further analyzing the genetic context, it was supposed that the original of *bla*_KPC-3_ in our work was a series of mutation of *bla*_KPC-2_.

**Conclusions:**

The emergence of a multidrug resistance IncP-2 megaplasmid and clonal transmission of *bla*_KPC-3_-producing *P. aeruginosa* in China underlined the crucial need for continuous monitoring of *bla*_KPC-3_ for prevention and control of its further dissemination in China.

**Supplementary Information:**

The online version contains supplementary material available at 10.1186/s12941-023-00577-z.

## Background

Due to the irrational use of antibiotics, the quantity of multidrug-resistant (MDR) bacteria [1] is on rise, and the largest proportion of them is *Enterobacteriaceae*. For infections caused by MDR bacteria, carbapenems are often the preferred treatment. Consequently, the appearance of carbapenem-resistant organisms (CRO) invalidates many commonly used antibiotics and leads to clinical treatment failure, which causes a high mortality rate in several regions, especially developing countries [2, 3]. The leading cause of CRO is carbapenem-hydrolyzing enzymes, which account for about 90%. The presence of the high-yield AmpC enzyme combined with the deletion of membrane pore proteins or the high expression of the excretion system accounts for a minor portion of CRO [4]. There are many types of carbapenemases, including KPC, NDM, IMP, VIM, OXA-48 and so on [5].

KPC, one of the carbapenemases family representatives, was first identified in *Klebsiella pneumoniae* from a hospital in North Carolina in 2001 [6]. Since then, different variants of *bla*_KPC_ genes have been identified globally [7–9]. KPC-3, a common type of KPC, which differs from KPC-2 with a His (272) -Tyr substitution [10, 11]. KPC-3 was initially reported in a New York Medical Center in 2004 and had been detected in Spain, Brazil, and Africa [12–14]. However, there have been few reports about KPC-3 in China until now [15, 16].

As an important pathogen causing nosocomial infections worldwide, *Pseudomonas aeruginosa* is known to become carbapenem-resistant by the overexpression of resistance-nodulation-division (RND) efflux systems, the absence of porin (OprD), AmpC-lactamase encoding and carbapenemase production [17]. This is one of the reasons for the high mortality of infections caused by *P. aeruginosa*. Of which, the acquisition of antimicrobial in *P. aeruginosa* mainly through the horizontal transfer of plasmids [18]. Currently, IncP incompatibility groups have been reported in *P. aeruginosa*, include IncP-1 to IncP-7 and IncP-9 to IncP-14 [19].

Herein, we identified *P. aeruginosa* isolates carrying *bla*_KPC-3_, and performed whole-genome sequencing (WGS). Meanwhile, this study reported the antimicrobial resistance profiles and genomics characterization of *bla*_KPC-3_-harboring *P. aeruginosa* with an IncP-2 megaplasmid.

## Materials and methods

### Clinical isolates and antimicrobial susceptibility testing

From March 2021 to December 2021, a total of 37 carbapenem-non-susceptible *P. aeruginosa* isolates were obtained from a teaching hospital of Zhejiang University. All isolates were inoculated on the agar plate and cultured at 37 °C for 18 h. Species were identified using matrix-assisted laser desorption ionization time-of-flight mass spectrometry (MALDI-TOF-MS) (Bruker, Bremen, Germany). Carbapenemases encoding genes including *bla*_KPC_, *bla*_NDM_, *bla*_OXA-48_, *bla*_VIM_, and *bla*_IMP_ were identified using polymerase chain reaction (PCR) and genomic sequencing, as described previously [20]. Susceptibility testing of polymyxin was performed by broth dilution method, and other antibiotics such as aztreonam, imipenem, meropenem, ceftazidime, levofloxacin, ciprofloxacin, gentamicin, piperacillin/tazobactam, cefepime, amikacin, ceftazidime/avibactam were performed by agar dilution method. Interpretation of susceptibility results was according to Clinical and Laboratory Standards Institute (CLSI) (https://clsi.org/) guidelines, except polymyxin which the susceptibility breakpoints were proposed by the European Committee on Antimicrobial Susceptibility Testing (EUCAST) (http://www.eucast.org) [21]. *P. aeruginosa* ATCC 27853 were used as a quality control standard.

### Location of ***bla***_KPC-3_ gene and transferability of plasmids carrying ***bla***_KPC-3_ gene

In order to analyze the homology of strains, we performed the PFGE experiments. The size and number of plasmids were identified by the S1-nuclease digestion and pulsed-field gel electrophoresis (S1-PFGE). After electrophoresis, the location of *bla*_KPC-3_ gene was determined by Southern blotting and hybridization with digoxigenin-labeled *bla*_KPC-3_ specific probe. *Salmonella* strain H9812 was used as a control strain and size marker [20].

The transferability of the plasmid carrying *bla*_KPC-3_ was verified by conjugation experiments using rifampin-resistant *P. aeruginosa* PAO1Ri and *E. coli* 600 as the recipient strain. Mueller–Hinton medium containing 300 mg/L rifampicin and 2 mg/L meropenem was used as the screening medium for transconjugants. The transconjugants were identified by MALDI-TOF-MS, and then the *bla*_KPC-3_ was screened by PCR to confirm whether the plasmids were successfully transferred [22].

### Whole-genome sequencing and plasmid analysis

Genomic DNA was extracted by using a Bacterial DNA Kit (QIAGEN, Hilden, Germany). Then the DNA was sequenced both on the Illumina NovaSeq 6000 (Illumina, San Diego, CA, United States) and Oxford Nanopore platforms (Oxford Nanopore Technologies, Oxford, United Kingdom) to obtain short-read data and long-read data, respectively. The complete genome sequence was assembled with Unicycler v0.4.7. The bacterial genome was annotated by the RAST server (https://rast.nmpdr.org/). Genotyping based on the analysis of internal fragments of seven housekeeping genes (*acsA*, *aroE*, *guaA*, *mutL*, *nuoD*, *ppsA* and *trpE*) was performed via the online service MLST (https://cge.cbs.dtu.dk/services/MLST/) [23]. Additionally, the acquired ARGs were detected by ResFinder 4.1 (https://cge.food.dtu.dk/services/ResFinder/), and the plasmid replicon type was identified by PCR [24]. The analysis of virulence factors was performed on VFDB website (http://www.mgc.ac.cn/cgi-bin/VFs/v5/main.cgi). The origin of transfers in DNA sequences of bacterial mobile genetic elements was identified by oriTfinder tool (https://tool-mml.sjtu.edu.cn/oriTfinder/oriTfinder.html). The transposon and insertion sequence were detected using the ISFinder database (http://www-is.biotoul.fr/). Finally, the comparison figures of the genetic context surrounding *bla*_KPC-3_ were generated by Easyfig2.0 software, and BLAST Ring Image Generator (BRIG) was used to generate the circular image of multiple plasmids comparisons.

### Nucleotide sequence accession numbers

The complete sequence of pLHL1-KPC-3 has been submitted to GenBank under accession no. CP099961.

### Biofilm formation assays

Biofilm formation was measured in accordance with the assays outlined in previous researches [25]. In a word, the bacterial overnight culture was diluted in LB and the 200 μL of the mixture was dispensed into the each well of 96-well plate. After static culture at 37℃ for 24 h, PBS was utilized to clean the microwells three times in order to eliminate all non-adherent bacteria. Methanol was used for fixation, and 0.1% crystal violet solution were added to each well for subsequent stain. After washing the plate with PBS three times and discarding the washing solution, 100 μL of DMSO were added to dissolve the crystal violet attached to the biofilm, and the plate should be incubated for 5 min. Finally, the OD (optical density) was measured at 590 nm. LB broth was used for the negative control. ODc is the mean OD of the negative control, ODs is the mean OD of samples. The experimental results were interpreted using the following criteria: ODc ≥ ODs: non biofilm formation, ODc < ODs ≤ 2*ODc: weak biofilm formation, 2*ODc < ODs ≤ 4*ODc: moderate biofilm formation, 4*ODc < ODs: strong biofilm formation.

## Results

### Isolation and identification of KPC-3-producing *P. aeruginosa*

Five KPC-3-producing *P. aeruginosa* strains were obtained from two different patients. The clinical characteristics of the strains are shown in Additional file 6: Table S1. The first strain of *bla*_KPC-3_-carrying *P. aeruginosa* LHL-1 was isolated from the sputum sample of patient 1 in the neurological ward on May 11, 2021. Two weeks later, we obtained another strain of *P. aeruginosa* LHL-11 from the sputum culture of patient 1 again. Three months later, while patient 1 was admitted to ICU, two drug-resistant strains LHL-20, LHL-28 were isolated. In contrast, LHL-37 strain was isolated from patient 2 in the neurological ward on October 9, 2021.

### Antimicrobial susceptibility profiles

The minimum inhibitory concentration (MIC) values of antibiotics for five isolates are shown in Table 1. All five isolates had similar resistance profiles, resistant to aztreonam, imipenem, meropenem, ceftazidime, levofloxacin, ciprofloxacin, gentamicin, piperacillin/tazobactam, cefepime, but sensitive to amikacin, ceftazidime/avibactam, polymyxin.

**Table 1:** Susceptibilities of the *P. aeruginosa* LHL-1, LHL-11, LHL-20, LHL-28, and LHL-37

	MIC (mg/L) of strain				
Antibiotics	LHL-1	LHL-11	LHL-20	LHL-28	LHL-37
Aztreonam	> 128	> 128	> 128	> 128	> 128
Imipenem	> 32	> 32	> 32	> 32	> 32
Meropenem	> 32	> 32	> 32	> 32	> 32
Ceftazidime	128	128	128	128	128
Levofloxacin	8	8	8	8	8
Ciprofloxacin	4	4	4	8	4
Amikacin	16	16	16	16	16
Gentamicin	8	8	8	8	8
Piperacillin/tazobactam	> 128	> 128	> 128	> 128	> 128
Cefepime	> 128	> 128	> 128	> 128	> 128
Ceftazidime/avibactam	8	8	8	8	8
Polymyxin B	2	1	2	1	1

### Biofilm formation assays

**Fig.1:**
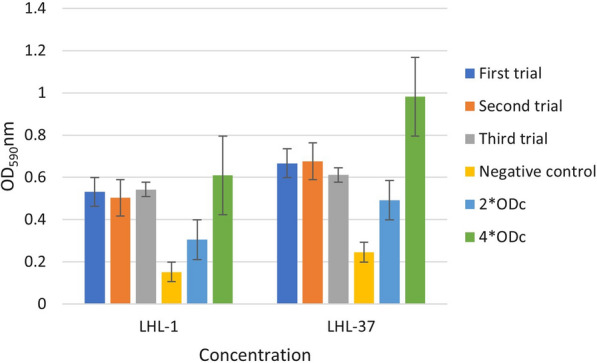
The biofilm biomass of LHL-1 and LHL-37

The LHL-1 from patient 1 and LHL-37 from patient 2 were selected for biofilm formation assay. The biofilm assay shown in Fig. 1 revealed that LHL-1 and LHL-37 had a moderate capacity for biofilm formation, with all mean OD values of trial groups greater than 2*ODc.

### Genomic characteristics of LHL-1

The results of PFGE showed the five strains were highly homologous (Additional file 5: Fig. S5). By comparing the whole-genome data, we found that the sequences of the five strains were highly consistent and all belonged to a novel sequence type ST1076, so the strain LHL-1 was selected for further genomics analysis. It was found that the genome consists of a 7,232,504 bp circular chromosome with an average G + C content of 65.5% and a 394,987 kb megaplasmid with 491 predicted coding regions. By further analyzing the genome sequence of LHL-1, there were many ARGs such as coding for aminoglycoside (*aph(3')-IIb*), ciprofloxacin (*crpP*), chloramphenicol (*catB7*), beta-lactam (*bla*_OXA-395_, *bla*_PAO_) and fosfomycin (*fosA*) encoding on the chromosome. Besides, only one carbapenem resistance gene, *bla*_KPC-3_, was encoded on the megaplasmid. Virulence profiles of LHL-1 mainly including *pilD* (Type IV pili biosynthesis), *chpA*, *pilG* (Type IV pili twitching motility related proteins) and *csrA* (Carbon storage regulator A), as in LHL-37.

### Characterization of megaplasmids harboring ***bla***_KPC-3_

**Fig. 2:**
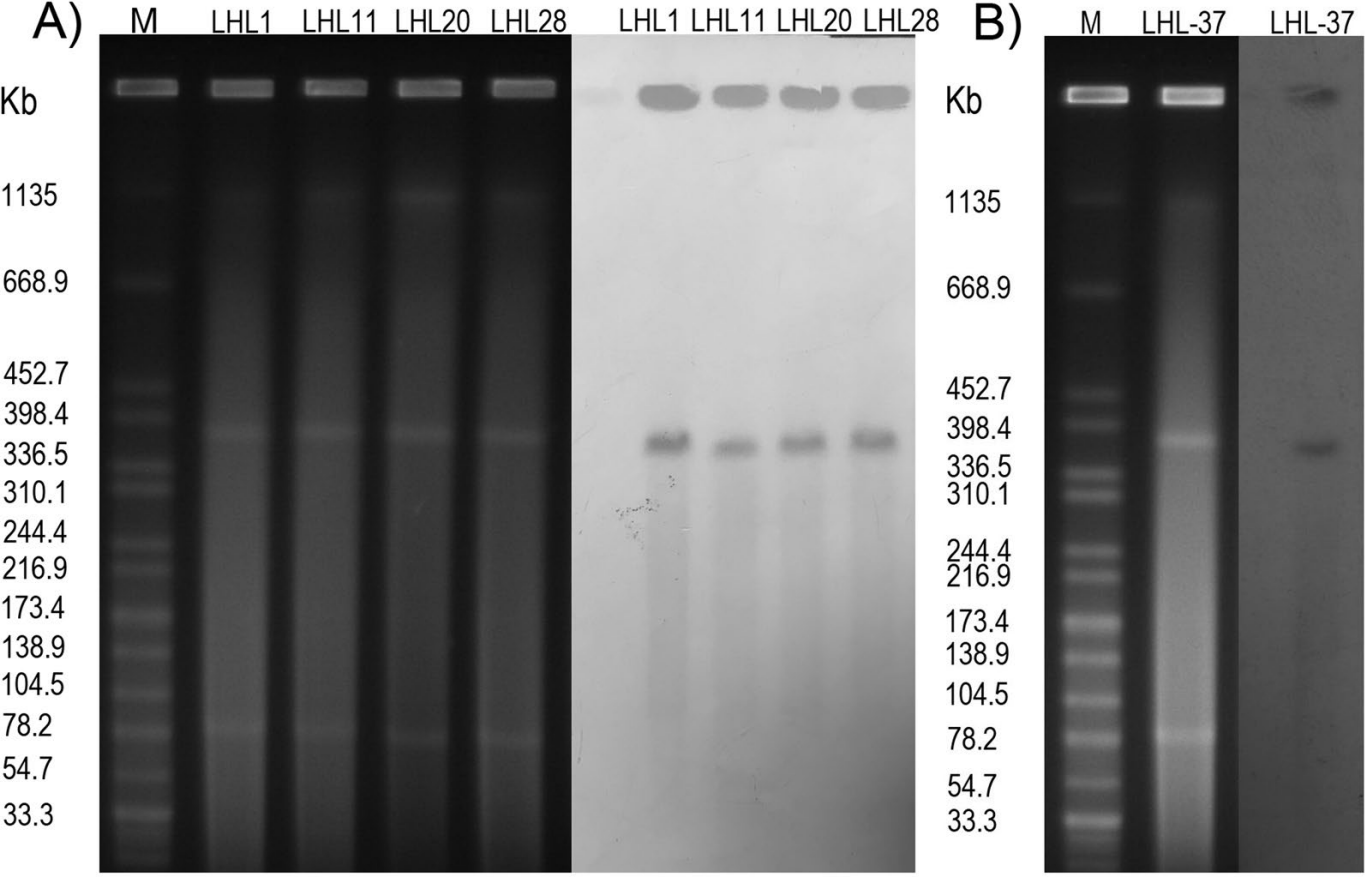
Plasmid profiles of all isolates. S1-PFGE and Southern blotting hybridization of (**A**) *P. aeruginosa* LHL-1, LHL-11, LHL-20, LHL-28, and (**B**) *P. aeruginosa* LHL-37. This image was a cropped version of the original picture

According to the S1-PFGE, it can be determined that the five isolates all harbor two plasmids with different sizes, and the results of Southern blotting revealed that *bla*_KPC-3_ gene was located on the megaplasmid with 394,987 bp in length (Additional files 1, 2, 3, 4 and Fig. 2). The *bla*_KPC-3_-harboring megaplasmids in isolates LHL-1 and LHL-37 are identical, containing 353 predicted open reading frames and a G + C content of 56.57%. Therefore, we chose the pLHL1-KPC-3 for further experiment.

**Fig. 3:**
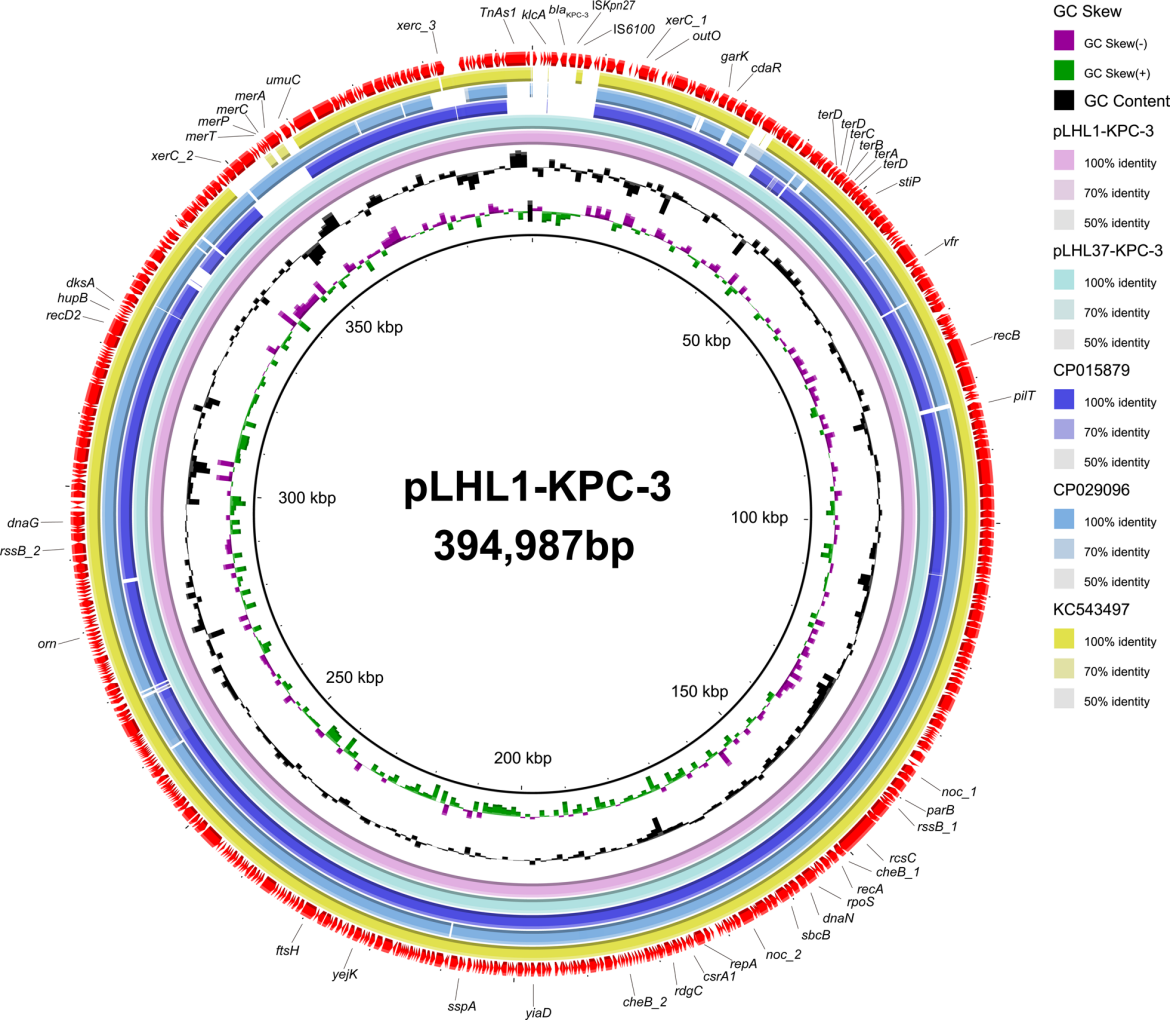
Comparative analysis of plasmids pLHL1-KPC-3 with the pLHL37-KPC-3, pRBL16 (CP015879), plasmid unnamed-2-AR439 (CP029096), and pOZ176 (KC543497)

We tried to transfer plasmid pLHL1-KPC-3 to the recipient strain *P. aeruginosa* PAO1Ri and *E. coli* 600. Nevertheless, no *bla*_KPC-3_-positive transformant was obtained in our study. Many functional genes were distributed on the skeleton megaplasmid pLHL-1-KPC-3, relating to replication and partitioning. By searching the core plasmid region against those in GenBank, the pLHL1-KPC-3 backbone showed 91% query coverage and 99% nucleotide identity to plasmid pRBL16 from *P. citronellolis* strain SJTE-3 (GenBank accession number CP015879), 94% query coverage and 100% nucleotide identity to pOZ176 which belonged to the IncP-2 group with IMP-9-mediated carbapenem resistance from *P. aeruginosa* strain PA96 (GenBank accession number KC543497), 92% query coverage and 98.27% nucleotide identity to plasmid unnamed-2 from *P. aeruginosa* strain AR439 (GenBank accession number CP029096) (Fig. 3). The information about the 20 related megaplasmids is shown in Table 2.

**Table 2:** Information about the related megaplasmid

Plasmid	Species	Size(bp)	Year of isolation	Source	Country	Genbank accession no
pRBL16	*P. citronellolis*	370338	2015	Sludge	China	CP015879
pOZ176	*P. aeruginosa*	500839	2000	Clinical	China	KC543497
plasmid unnamed2-AR439	*P. aeruginosa*	437392	NA	Clinical	NA	CP029096
pPAG5	*P. aeruginosa*	513322	2016	Clinical	China	CP045003
pPABL048	*P. aeruginosa*	414954	2001	Clinical	USA	CP039294
plasmid unnamed2-AR0356	*P. aeruginosa*	438531	NA	Clinical	USA	CP027170
pBM413	*P. aeruginosa*	423017	2012	Clinical	China	CP016215
p1-P19E3	*P. koreensis*	467568	2014	Environment	Switzerland	CP027478
pBT2101	*P. aeruginosa*	439744	2014	Clinical	Thailand	CP039991
p12939-PER	*P. aeruginosa*	496436	NA	Clinical	China	MF344569
pBT2436	*P. aeruginosa*	422811	2013	Clinical	Thailand	CP039989
pBM908	*P. aeruginosa*	395774	2018	Clinical	China	CP040126
plasmid unnamed3-AR441	*P. aeruginosa*	438529	NA	Clinical	USA	CP029094
pJB37	*P. aeruginosa*	464804	2008	Clinical	Portugal	KY494864
p727-IMP	*P. aeruginosa*	430,173	NA	Clinical	China	MF344568
pA681-IMP	*P. aeruginosa*	397519	NA	Clinical	China	MF344570
pTTS12	*P. putida*	583900	1989	Soil	Netherlands	CP009975
pR31014-IMP	*P. aeruginosa*	374000	NA	Clinical	China	MF344571
plasmid1-RW109	*P. aeruginosa*	555265	NA	Industrial	NA	LT969519
pSY153-MDR	*P. putida*	468170	2012	Clinical	China	KY883660

**Fig. 4:**
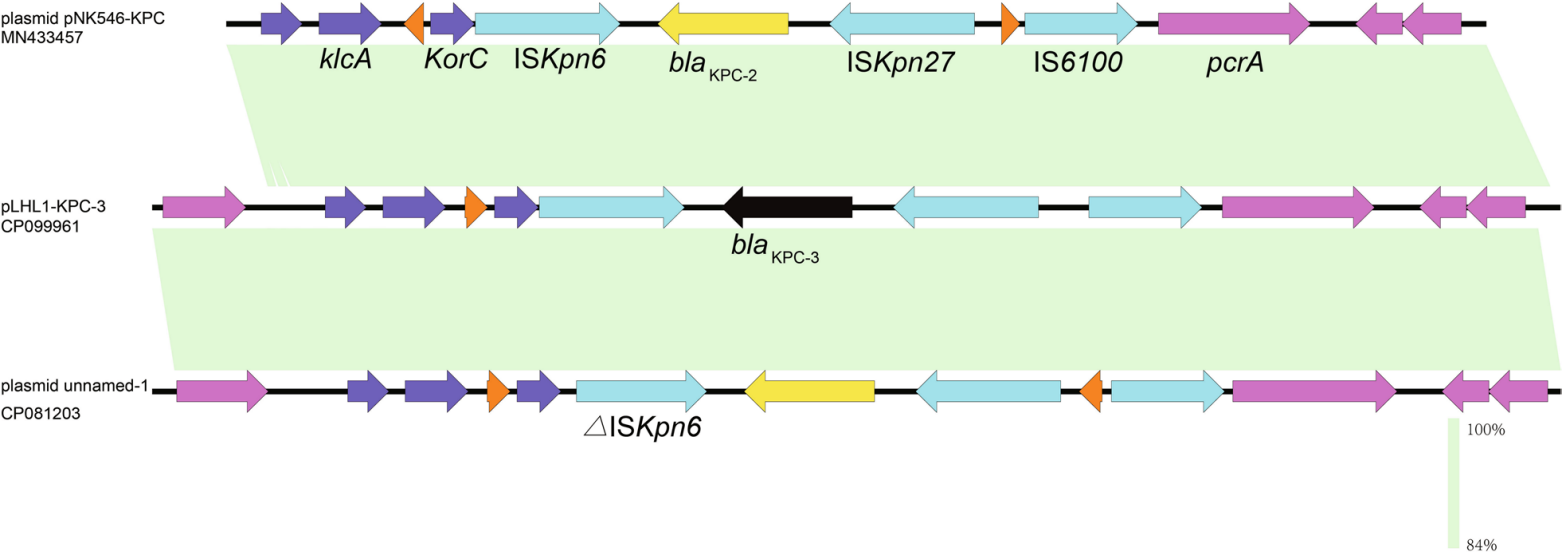
The genetic context of *bla*_KPC-3_ gene on plasmid pLHL1-KPC-3, plasmid pNK546-KPC (MN433457), and plasmid unnamed-1 (CP081203). Open reading frames (ORFs) are shown as arrows and indicated according to their putative functions. Black and yellow indicates antimicrobial resistance genes and blue indicates genes related to mobile elements. The pink represents other functional genes. Regions with a high degree of homology are indicated by light green shading

Genetic environment analysis showed that IS*Kpn6* (upstream), IS*Kpn27* (downstream) and several mobile elements including IS*6100* and Tn*As1* are densely distributed around *bla*_KPC-3_. Notably, a conserved structure sequence (IS*6100*-IS*Kpn27*-*bla*_KPC-3_-IS*Kpn6*-*korC*-*klcA*) was observed in the plasmid structure (Fig. 4). The genetic environment surrounding the *bla*_KPC-3_ in pLHL1-KPC-3 was related to the region of plasmid unnamed-1 from *P. aeruginosa* strain P9W (> 99% identities and > 99% query coverage; GenBank accession number CP081203) and plasmid pNK546-KPC from *P. aeruginosa* strain PAB546 (> 99% identities and > 99% query coverage; GenBank accession number MN433457), isolated from China.

## Discussion

On the list for research and development of new antibiotics reported by the World Health Organization in 2017, carbapenem-resistant *P. aeruginosa* (CRPA) was ranked as one of the top antibiotic-resistant bacteria [26]. In contrast to *K. pneumoniae*, the incidence of plasmid-mediated *bla*_KPC-3_ was comparatively less frequent in *Pseudomonas spp*. To date, there have been no reports of KPC-3-producing *P. aeruginosa* infection in China. Using the keywords “*pseudomonas*”, “KPC-3”, and “China” to search the PubMed database (last accessed on 5 Dec. 2022), it retrieved zero results. Here, we collected the *bla*_KPC-3_ producing *P. aeruginosa* isolates, investigated the molecular characterization and genetic background of the *bla*_KPC-3_-harboring megaplasmid.

Over the period of five months, five identical strains of drug-resistant bacteria were isolated from two distinct patients over five months. Simultaneously, neither patient had a history of foreign residency. Considering the similarity of the genome and plasmid characteristics of the five bacterial strains, we inferred that clonal transmission occurred between the two patients. All five strains of *P. aeruginosa* carrying *bla*_KPC-3_ with a novel sequence type ST1076. Up to now, the* bla*_KPC-3_ has been detected in various ST-type strains, such as ST11 [27], ST147 [28], and ST258 [29]. To our knowledge, the study about ST1076 is limited, except one research has reported that ST1076 is associated with epidemiologically well-described outbreaks in burn units [30]. There is no obvious relationship between the new clone and the spread of *bla*_KPC-3_. However, the new ST type reminded us that mutations occurred in *P. aeruginosa* which deserve our attention. Biofilm forming ability partly reflects the virulence ability of bacteria. In the study, LHL-1 and LHL-37 both exhibited a moderate ability to form biofilms and virulence profiles were also identical. The contained virulence factors were primarily contributed to the biofilm formation and colonization in infection [31]. Infection caused by *P. aeruginosa* with certain biofilm capacity poses a threat to clinical treatment.

To the best of knowledge, the *bla*_KPC_ gene can be either plasmid-mediated or on the chromosome of the host. Many types of plasmid replicons which was relative to *Enterobacteriaceae* carried the *bla*_KPC_, including IncX3, IncFrepB, IncQ and IncFIIK2-FIB [32, 33]. Unlike the common plasmid replicon types, *bla*_KPC_-harboring plasmid in *P. aeruginosa* often could be recognized as other different incompatibility groups, or could not be classified. A previous study reported a non-conjugative but mobilizable IncP-6-type resistance plasmid p10265-KPC in China [19]. Hu et al. described a novel plasmid carrying carbapenem-resistant gene *bla*_KPC-2_ in *P. aeruginosa* [34]. Moreover, there were many researches represented megaplasmids family carrying different ARGs in *Pseudomonas* spp. [35, 36]. Our study firstly identified a *bla*_KPC-3_-carrying IncP-2 megaplasmid. There have been several reports on ARGs-associated IncP-2 megaplasmids which were commonly found in the isolates of *P. aeruginosa* [37, 38]. Currently, most of the reported ARGs carried by IncP-2 megaplasmid encoded metallo-β-lactamases (MBLs),such as IMP and VIM. [39]. The discovery of *bla*_KPC-3_-carrying IncP-2 megaplasmid complements a novel example of IncP-2 megaplasmid family and its evolution, which deserves further investigation.

The oriTfinder result showed there no Origin site of DNA transfer (*oriT*), *Relaxase*, Type IV coupling protein (T4CP) and Type IV secretion system (T4SS) were found in the pLHL-KPC-3. The lack of conjugal transfer gene regions in pLHL-1 backbone was consistent with the fact that the vitro conjugation experiments were unsuccessful [40, 41]. By investigating the backbone structure of pLHL-1-KPC-3, the *repA* gene encodes the plasmid replication initiator protein and the *parB* encodes the protein that binds to the centromere-like sites, respectively. The *ter* gene was associated with the tellurite resistance as part of the megaplasmid family core genome. The *pliT* gene encodes PilT domain-containing protein. The *merP* was responsible for mercuric transport protein periplasmic component while the *merT* gene was related with mercuric transport protein [42].

Unlike the previous literature that *bla*_KPC_ gene was embedded on Tn*4401* generally, a member of the Tn3 family transposon involved in gene acquisition and dissemination, *bla*_KPC-3_ gene with the linear structure IS*6100*-IS*Kpn27*-*bla*_KPC-3_-IS*Kpn6*-*korC*-*klcA* in this work was found adjacent to another Tn*3* family transposase Tn*As1* [43, 44]. IS*6100*, belonging to the IS*6* family members, is also one of the insertion sequences closely related to ARGs and can mediate sequence transfer and recombination between chromosomes or plasmids in different bacteria [45]. BLAST results showed that this region IS*6100*-IS*Kpn27*-*bla*_KPC-3_-IS*Kpn6*-*korC*-*klcA* was highly similar to those in the *bla*_KPC-2_-harboring element which played a key role in its transmission [46]. It was reasonable to presume that the sequence may also be involved in the transmission of *bla*_KPC-3_. By comparing the closely resemble plasmid backbone of pLHL1-KPC-3 and the other two plasmids, the major difference was the different ARGs variants they carried (Fig. 3). These results suggested the megaplasmid can capture diverse segment of ARGs based on the formation of backbone and then transfer to different host strains.

Although the isolates of KPC-3-producing *P. aeruginosa* in our work were not susceptible to multiple antibiotics, they remained susceptible to ceftazidime-avibactam (CAZ-AVI). In 2019, CAZ/AVI was used for clinical treatment in China. However, the resistance to CAZ-AVI has emerged and is more likely to occur in *bla*_KPC-3_-producing strains. Shields has reported the emergence of CAZ-AVI resistance in KPC-3-carbapenem-resistant *K. pneumoniae* during the treatment [47]. Thus, CAZ/AVI should be applied cautiously in the clinical treatment of infections caused by KPC-3-producing *P. aeruginosa*.

## Conclusion

To our knowledge, this is the first report about KPC-3 producing *P. aeruginosa* in China. And our work revealed the characterization of an IncP-2 megaplasmid carrying *bla*_KPC-3_. This study reminded us that *bla*_KPC-3_ has gradually appeared in China with related to various bacterial species. Furthermore, it contributes to study the significance of megaplasmids in the evolution and resistance acquisition by *P. aeruginosa*. At the same time, we should also strengthen surveillance of CRPA spread in hospitals to prevent outbreaks of nosocomial infections.

## Supplementary Information

**Additional file 1.** The original gel picture of *P. aeruginosa* LHL1, LHL11, LHL20 and LHL28.

**Additional file 2.** The original gel picture of *P. aeruginosa* LHL37.

**Additional file 3.** The original blot image of *P. aeruginosa* LHL1, LHL11, LHL20 and LHL28.

**Additional file 4.** The original blot image of *P. aeruginosa* LHL37.

**Additional file 5.** The PFGE result of five KPC-3-producing *P. aeruginosa* strains.

**Additional file 6: Table S1.** The clinical characteristics of the clinical strains.

## Data Availability

The complete sequence of pLHL1-KPC-3 has been submitted to GenBank under accession no. CP099961.

## Abbreviations

KPC: *Klebsiella pneumoniae* Carbapenemase
ARGs: Antimicrobial resistance genes
AST: Antimicrobial susceptibility testing
S1-PFGE: S1-nuclease pulsed-field gel electrophoresis
MDR: Multidrug-resistant
CRO: Carbapenem-resistant organisms
RND: Resistance-nodulation-division
WGS: Whole-genome sequencing
PCR: Polymerase chain reaction
MALDI-TOF-MS: Matrix-assisted laser desorption ionization time-of-flight mass spectrometry
CLSI: Clinical and Laboratory Standards Institute
EUCAST: European Committee on Antimicrobial Susceptibility Testing
MIC: Minimum inhibitory concentration
CRPA: Carbapenem-resistant *P. aeruginosa*
CAZ-AVI: Ceftazidime-avibactam
