# Supplementary figures and images for: Emergence and clonal dissemination of KPC-3-producing Pseudomonas aeruginosa in China with an IncP-2 megaplasmid
Source: Ann Clin Microbiol Antimicrob. 2023 Apr 29;22:31. doi: 10.1186/s12941-023-00577-z (PMC10149002; doi:10.1186/s12941-023-00577-z)

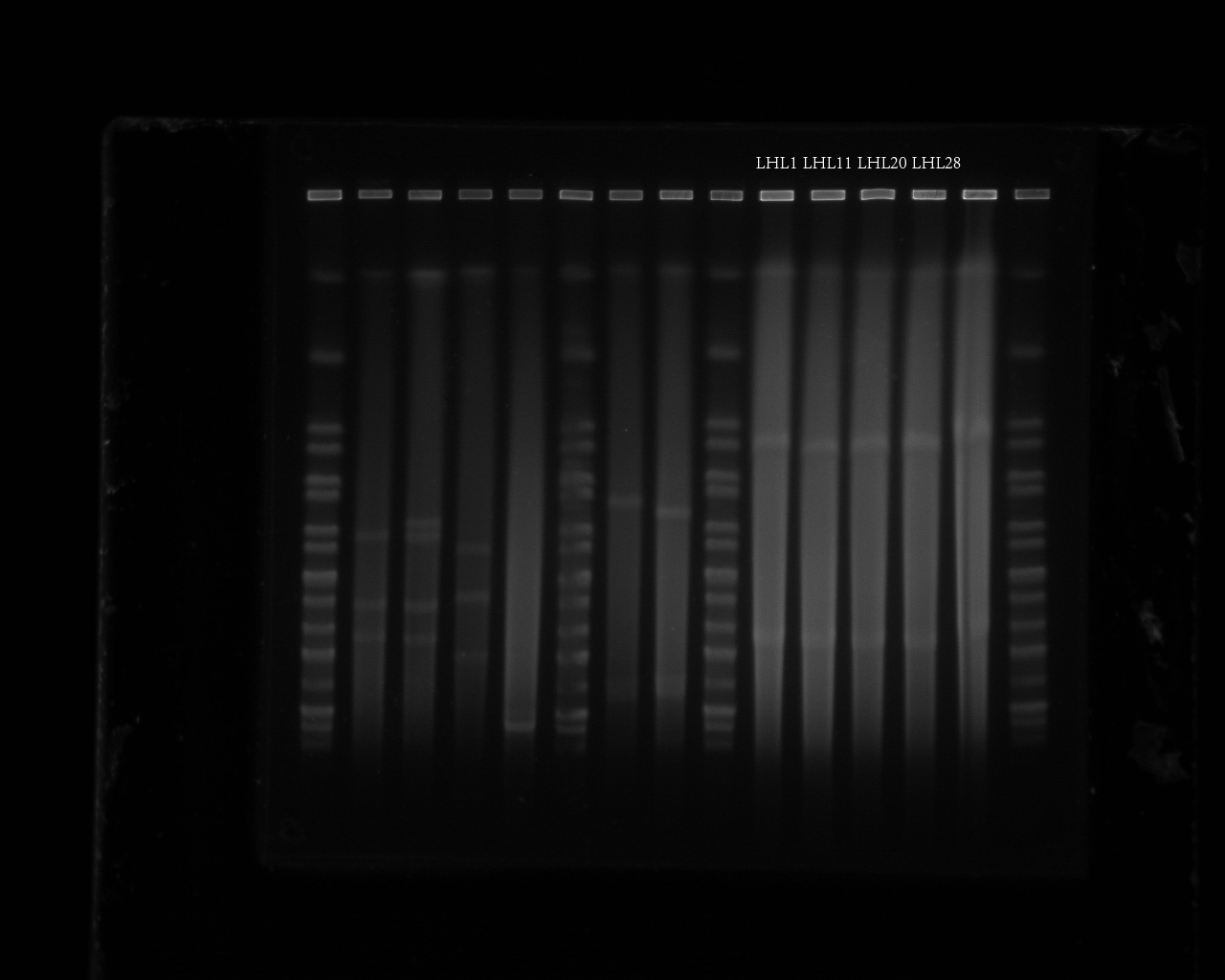

Supplement: Supplementary file 1 — Additional file 1. The original gel picture of P. aeruginosa LHL1, LHL11, LHL20 and LHL28. [file 12941_2023_577_MOESM1_ESM.jpg]

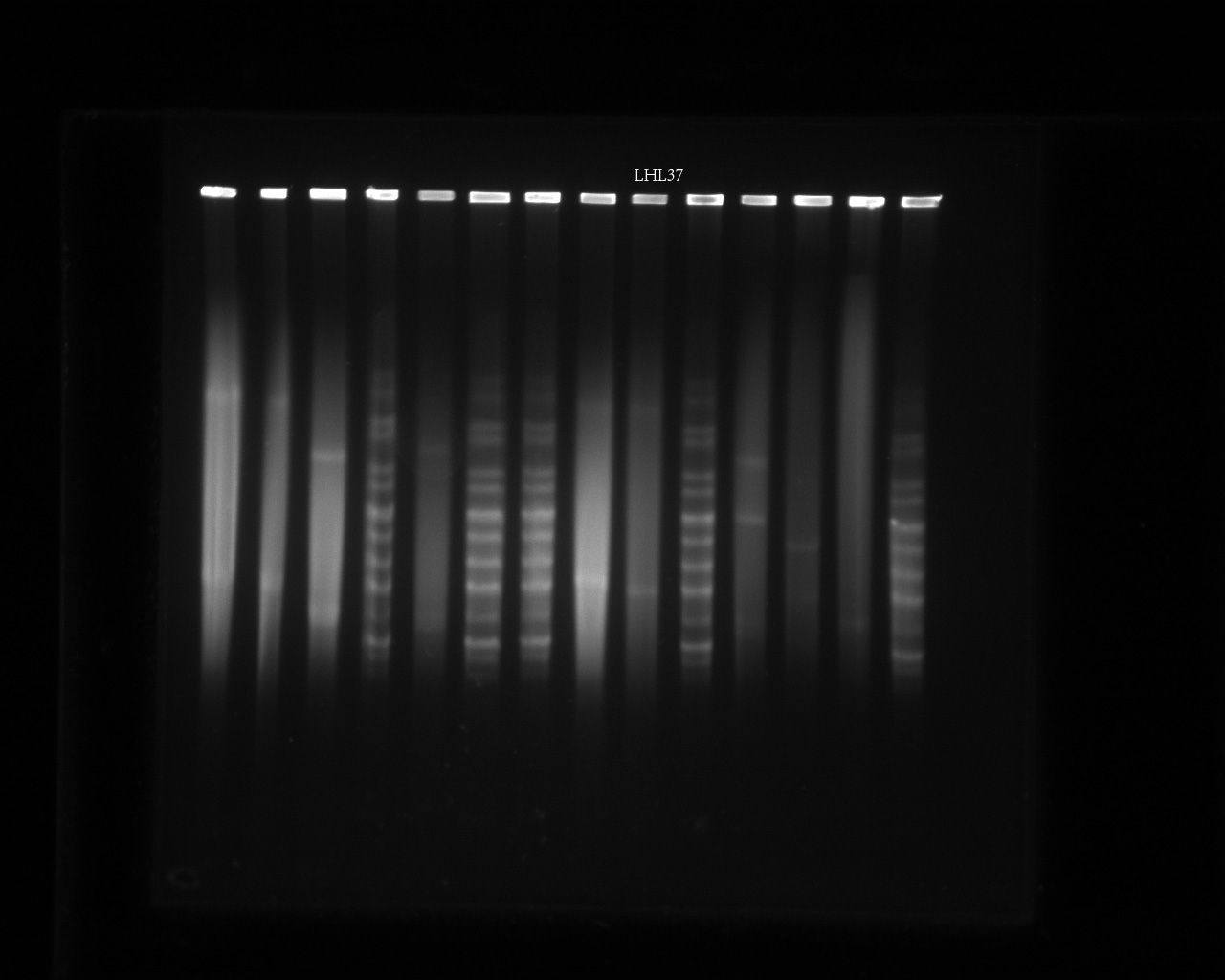

Supplement: Supplementary file 2 — Additional file 2. The original gel picture of P. aeruginosa LHL37. [file 12941_2023_577_MOESM2_ESM.jpg]

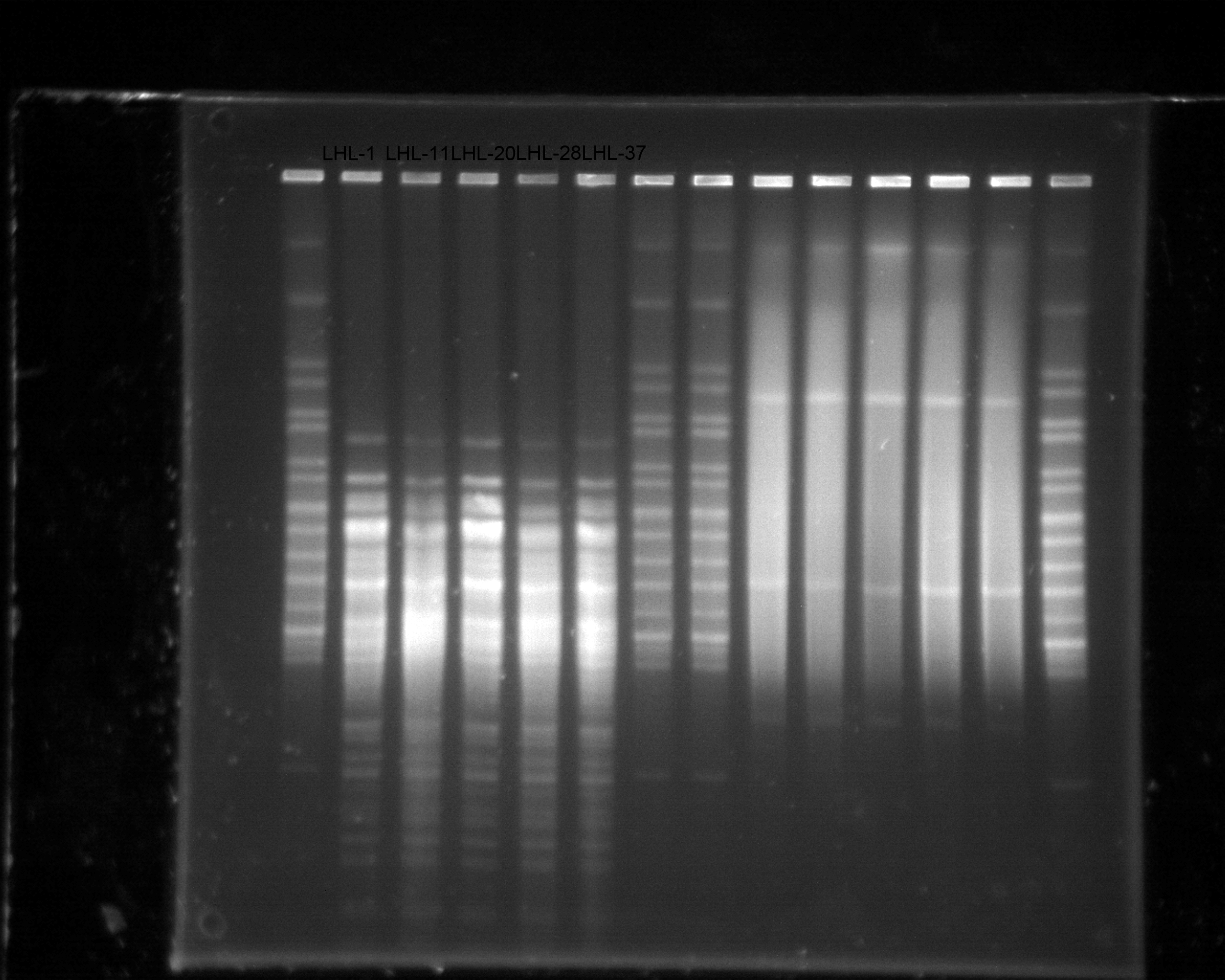

Supplement: Supplementary file 5 — Additional file 5. The PFGE result of five KPC-3-producing P. aeruginosa strains. [file 12941_2023_577_MOESM5_ESM.tif]
