# Supplementary material for: Emergence and clonal dissemination of KPC-3-producing Pseudomonas aeruginosa in China with an IncP-2 megaplasmid
Source: Ann Clin Microbiol Antimicrob. 2023 Apr 29;22:31. doi: 10.1186/s12941-023-00577-z (PMC10149002; doi:10.1186/s12941-023-00577-z)
Supplement: Supplementary file 6 — Additional file 6: Table S1. The clinical characteristics of the clinical strains. [file 12941_2023_577_MOESM6_ESM.docx]

| **Table S1** The clinical characteristics of the clinical strains^α^ | | | | | | | | |
| --- | --- | --- | --- | --- | --- | --- | --- | --- |
|  | Age  (years), sex | Diagnosis | Ward | Strain | Isolation date | Specimen | ST | KPC |
| Case 1 | 71, F | ARF  PD | Neurosurgery | LHL-1 | 11 May  2021 | sputum | 1076 | KPC-3 |
|  |  |  | Neurosurgery | LHL-11 | 26 May  2021 | sputum | 1076 | KPC-3 |
|  |  |  | ICU | LHL-20 | 15 August  2021 | sputum | 1076 | KPC-3 |
|  |  |  | ICU | LHL-28 | 27 August  2021 | sputum | 1076 | KPC-3 |
| Case 2 | 68, M | PTE | Neurosurgery | LHL-37 | 9 October  2021 | sputum | 1076 | KPC-3 |
| ^α^ARF, acute respiratory failure; PD, persistent diarrhea; PTE, Pulmonary Thrombo embolism | | | | | | | | |
